# Genome-wide characterization of the *2OGD-Hs* gene family in apple reveals a GA-metabolic gene, *MdGA2ox1b*, confers stress tolerance via MdHY5 regulation

**DOI:** 10.3389/fpls.2026.1911733

**Published:** 2026-10-15

**Authors:** Shuangxun Ma, Danni Zhang, Chao Yang

**Affiliations:** 1 School of Future Transportation, Chang’an University, Xi’an, China; 2 State Key Laboratory for Crop Stress Resistance and High-Efficiency Production/Shaanxi Key Laboratory of Apple, College of Horticulture, Northwest A&F University, Yangling, Shaanxi, China

**Keywords:** abiotic stress, apple, hormone metabolism, Md2OGD-Hs, MdHY5

## Abstract

**Introduction:**

The 2-oxoglutarate/Fe(II)-dependent dioxygenase hormone-related subfamily (2OGD-H), a key branch of the 2OGD superfamily, plays a crucial role in plant growth, development, stress adaptation and in the biosynthesis and metabolic regulation of phytohormones. However, despite functional studies of several individual *2OGD-H* genes in apple (*Malus domestica*), a comprehensive genome-wide identification and systematic characterization of this subfamily remains lacking.

**Methods:**

In this study, we systematically identified 2OGD family members in the apple genome using conserved domain searches and phylogenetic analyses. Gene duplication, promoter cis-elements, and transcriptomic expression patterns were analyzed. The candidate gene *MdGA2ox1b* was functionally validated via overexpression/silencing in apple calli under salt, cold, and heat stress conditions. The direct regulatory relationship with the upstream transcription factor MdHY5 was verified using yeast one-hybrid, EMSA, GUS, and dual-luciferase assays.

**Results:**

This current study systematically identified 257 members of the *2OGD* family in apple designated as Md2OGDs, which also included 58 *Md2OGD-Hs*. The *Md2OGD-Hs* were classified into nine evolutionary clades based on their conserved 2OG-FeII_Oxy and DIOX_N domains as well as clade-specific motifs. The study also showed that the *Md2OGD-Hs* expanded primarily from the whole-genome duplication events, and were closely related to *2OGD-Hs* in grapes (*Vitis vinifera*) than in rice (*Oryza sativa*). The *Md2OGD-Hs* consisted of 23 types of cis-acting elements associated with light responsiveness, hormone signaling, and stress adaptation, suggesting a multidimensional regulatory network in these genes. The transcriptomic data also demonstrated the broad regulatory roles of *Md2OGD-Hs* in apple tissue development, with 26 genes notably linked to fruit ripening. Through integrated analysis of multiple abiotic stress transcriptomes, we showed that *MdGA2ox1b* gene responds to various abiotic stresses and resides in the cytoplasm and nucleus, with a weak expression in the roots and high expression in the flowers. This gene also significantly enhances tolerance to salt, cold and heat stress in apple calli by activating antioxidant enzymes. MdHY5 directly bound its promoter and activated its expression, especially under cold stress.

**Discussion:**

This study elucidates the evolutionary dynamics and biological roles of *Md2OGD-Hs*, and provides a theoretical foundation and genetic resources for unravelling molecular mechanisms underlying stress resistance in apple.

## 1 Introduction

The 2-oxoglutarate/Fe(II)-dependent dioxygenase (2OGD) superfamily is one of the largest enzyme families in plant genomes and is ubiquitously present in plants (De Carolis and De Luca, 1994; Martens et al., 2010), where it regulates phytohormone metabolism, drives secondary metabolite biosynthesis, and orchestrates responses to environmental stresses through oxidative catalysis (Farrow and Facchini, 2014). The *2OGD* genes encode non-heme iron-containing oxidases that bind Fe^2+^ ions through the conserved 2OG-FeII_Oxy domain (PF03171). These enzymes utilize 2-oxoglutarate (2OG), ascorbate and catalase to oxidize 2OG into succinate and carbon dioxide, while catalyzing diverse oxidative reactions such as hydroxylation, methylation, demethylation, and epimerization (Martinez and Hausinger, 2015; Nadi et al., 2018; Ge et al., 2021). As critical regulators of plant metabolic networks, several members of the *2OGD* gene family have been characterized in multiple species, including 130 in *Arabidopsis thaliana*, 114 in *Oryza sativa*, 159 in *Solanum lycopersicum*, and 163 members in *Vitis vinifera* (Shu et al., 2007; Swarbreck et al., 2008; Ding et al., 2020; Gao et al., 2022). Based on conserved domains and phylogenetic relationships, the *2OGD* family is divided into three clades, including Dioxygenase Clade A (DOXA), Dioxygenase Clade B (DOXB), and Dioxygenase Clade C (DOXC) (Xu and Song, 2017). The DOXA clade primarily comprises alpha-ketoglutarate-dependent dioxygenase homologs (ALKBHs) involved in nucleic acid alkylation repair and epigenetic regulation (Falnes et al., 2002), while the DOXB clade encodes prolyl 4-hydroxylases (P4Hs) responsible for proline hydroxylation in cell wall proteins (Ozer and Bruick, 2007). On the other hand, the largest clade, DOXC, plays expansive roles in secondary metabolite biosynthesis and hormone metabolism (Clifton et al., 2006).

Members in the DOXC clade involved in the biosynthesis and metabolism of plant endogenous hormones are referred to as 2-oxoglutarate/Fe(II)-dependent dioxygenase hormone-related subfamily (2OGD-H) (Zhang et al., 2024a). The core members of this family include C19-gibberellin 2-oxidase (C19-GA2ox), C20-gibberellin 2-oxidase (C20-GA2ox), gibberellin 20-oxidase (GA20ox), gibberellin 3β-hydroxylase (GA3ox), 1-aminocyclopropane-1-carboxylic acid oxidase (ACO), indole-3-acetic acid oxidase (DAO), lateral root branching oxidase (LBO), jasmonic acid hydroxylase (JOX), and powdery mildew-related dioxygenase and dike-like oxygenase (DMR6/DLO). These 2OGD-H members play a central role in plant growth and development by dynamically regulating endogenous hormones. They also precisely integrate multiple abiotic stress signals to drive adaptive responses. For instance, transient silencing of *NtJOX3/5* in tobacco and knockout of its homologous gene *OsJOX3/4* in rice both increased endogenous jasmonic acid content, indicating the species-conserved negative regulation of *JOX3/5* on plant drought tolerance (Zhang et al., 2024a). In cucumber, *CsGA20ox* and *CsGA3ox* alleviate the inhibitory effect of salt stress on seed germination (Zhang et al., 2014), while overexpression of *GhACO106* enhances salt tolerance in Arabidopsis seedlings (Wei et al., 2021). The rice mutant *osga2ox6* exhibits significantly higher stress tolerance and yield compared to the wild type (Lo et al., 2017). Similarly, the potato *stdmr6–1* mutant shows stronger early resistance to *Fusarium* wilt and significantly improved salinity and drought tolerance compared to the wild type (Karlsson et al., 2024).

As a globally cultivated perennial fruit tree of significant economic value, apple faces multifaceted challenges during cultivation. The *Md2OGD-H* gene family plays a pivotal regulatory role in apple growth and development. Previous studies have revealed that *MdGA2oxs* and *MdGA20oxs* may negatively regulate floral bud formation through the gibberellin signaling pathway (Zuo et al., 2018; Zhang et al., 2023a), while *MdGA20oxs* may delay fruit abscission by modulating cell wall degradation in the abscission zone (Sriskantharajah et al., 2021). In addition, *MdACOs*, which are key components of ethylene biosynthesis, have been characterized during the fruit ripening processes (Gorny and Kader, 1996). *Md2OGD-Hs* also participate in both biotic and abiotic stress defense networks. For instance, *MdACO1* promotes light-induced anthocyanin biosynthesis (Yu et al., 2022), whereas *MdDMR6/DLOs* negatively regulate resistance to *Glomerella cingulata* by degrading salicylic acid (Shan et al., 2021; Pei et al., 2024). Notably, members of the GA2ox clade, whose promoter regions are rich in hormone-responsive and stress-related cis-elements, play a central role in maintaining GA homeostasis and balancing plant growth with abiotic stress tolerance. They function by inactivating bioactive gibberellins via 2β-hydroxylation and are widely induced by various stresses, including salinity, drought, low temperature, and heat (Shi et al., 2019; Zhang et al., 2024b). For instance, the apple *MdGA2oxs* genes are predominantly expressed in shoot tips, respond to exogenous GA3 treatment, and exhibit altered expression patterns under water deficit and salt stress (Zhang et al., 2023a). *MdGA2ox7* from apple enhances both salt and cold tolerance in apple calli, while simultaneously promoting anthocyanin accumulation (Yan et al., 2024).

HY5 (ELONGATED HYPOCOTYL 5), a basic leucine zipper (bZIP) transcription factor, has been well documented for its central role in plant light signaling. More recently, however, it has been positioned as a key node in the low-temperature tolerance network (Gangappa and Botto, 2016). In *Arabidopsis*, HY5 was shown to promote anthocyanin biosynthesis under cold stress, thereby suppressing ROS accumulation (Catalá et al., 2011). In tomato, SlFHY3 and SlHY5 were reported to act synergistically by integrating myo-inositol and light signals to enhance cold tolerance (Wang et al., 2022). Natural variation in the A-box motif of the maize (*Zea mays*) COOL1 promoter was found to alter HY5 binding affinity, which underlies the differential cold tolerance observed across maize varieties at contrasting latitudes (Zeng et al., 2025). HY5 has also been implicated in phytohormone-mediated cold acclimation. Strigolactones, for instance, were shown to promote HY5-dependent autophagy and facilitate the degradation of ubiquitinated proteins under low temperature in tomato (Chi et al., 2025). In apple, HY5 was demonstrated to tip the balance between growth and cold tolerance by significantly lowering the IAA/ABA ratio during cold stress (Liu et al., 2025).

While several individual *2OGD-H* genes and specific subfamilies have been investigated in apple (Zhang et al., 2023a; Yan et al., 2024), a comprehensive genome-wide analysis encompassing the entire hormone-related *2OGD-H* subfamily remains lacking, and its evolutionary characteristics and potential roles in abiotic stress responses remain incompletely understood. Therefore, this study comprehensively identified the apple *2OGD* gene family and systematically explored its sequence characteristics, evolutionary relationships, and gene duplication events. The study also integrated transcriptomic data to analyze tissue-specific expression patterns and expression modes under salt, drought, cold, and heat stress conditions. Based on the above analysis, a candidate gene, *MdGA2ox1b*, responsive to all four stresses was identified. Functional characterization revealed that overexpression of *MdGA2ox1b* significantly enhanced the drought, cold, and heat tolerance of apple calli. Further investigation showed that this gene is directly regulated by the low-temperature-induced transcription factor HY5 and is involved in the HY5-mediated establishment of cold tolerance in apple. These findings provide new insights into the molecular mechanisms of hormone-stress co-regulation in apples and offer theoretical support and evidence for apple stress-resistant genetic improvement and molecular breeding.

## 2 Materials and methods

### 2.1 Plant materials

Gene expression patterns were analysed in root, stem, leaf, flower, and mature fruit tissues of the apple cultivar ‘Golden Delicious’. Apple calli derived from the ‘Orin’ cultivar were used for genetic transformation, stress-induced expression profiling, and functional validation. All apple calli were maintained on MS solid medium supplemented with 1.5 mg/L of 2, 4-dichlorophenoxyacetic acid and 0.4 mg/L of 6-benzylaminopurine, adjusted to pH=5.8-6.0. Subculture was performed every 30 days in darkness in an incubator at 25 °C (Yang et al., 2025). *Nicotiana benthamiana* plants were grown in a controlled environment at 25 °C with a 16/8 h light/dark cycle, and their leaves were used for subcellular localization when the plants were 4 weeks old.

### 2.2 Identification, multiple sequence alignment, and phylogenetic analysis of apple 2OGD proteins

The 2OGD protein family in apple was identified using the GDDH13 v1.1 genome database (Daccord et al., 2017), with the 130 reported Arabidopsis 2OGD proteins as queries (Kawai et al., 2014). Candidate proteins were screened using the BLASTP program. A local HMMER 3.0 analysis was performed using the hidden Markov model (HMM) profile of the 2OG-FeII_Oxy structural domain (PF03171) from the InterPro database for further refinement (Mulder and Apweiler, 2007; Finn et al., 2011). Proteins without the 2OG-FeII_Oxy domains were excluded using NCBI Batch CD-Search and SMART. Partial members were systematically named according to the nomenclature rules of their Arabidopsis orthologs. Subsequently, TBtools (v2.154) Protein parameter calculator module was used to calculate the fundamental physicochemical properties, including amino acid length, molecular weight, isoelectric point (pI), instability index, aliphatic index and grand average of hydropathicity (Chen et al., 2023). Subcellular localization predictions were performed using the Plant-mPLoc online platform (Chou and Shen, 2010). The information on the *Md2OGDs* members is provided in **Supplementary Table S1**. Multiple sequence alignment was conducted using the E-INS-i algorithm available on the MAFFT online server. Phylogenetic analysis was conducted using the Maximum Likelihood method in MEGA 12 with 1000 bootstrap replicates for node support validation (Kumar et al., 2024), followed by topological optimization and visualization through the iTOL (Letunic and Bork, 2021).

### 2.3 Conserved domains and motif analysis, gene structure prediction, and cis-acting element analysis

The conserved domains of Md2OGD-H members are presented in **Supplementary Table S2**. The conserved motifs (**Supplementary Table S3**) of Md2OGD-H members were identified using the MEME Suite online tool with parameters set to 20 motifs (Bailey et al., 2015). Gene structure prediction was performed based on the apple GFF3 file (Daccord et al., 2017), while the promoter sequences of *Md2OGD-H* members were batch-extracted using TBtools (v2.154). The cis-acting elements (**Supplementary Table S4**) were predicted through the PlantCARE database and visualized using TBtools (v2.154) (Lescot et al., 2002).

### 2.4 Gene localization, collinearity analysis, and Ka/Ks calculation

The genomic positions of *Md2OGD-Hs* were retrieved from the apple GFF3 file and visualized using TBtools (v2.154). The GFF3 annotation files and genomic sequences of *Arabidopsis*, tomato, grape and rice were obtained from the Ensembl Plants database (Bolser et al., 2017). Intra- and interspecies collinearity analyses (**Supplementary Table S5**, **S6**) were conducted on the sequences and visualized using the One Step MCScanX-SuperFast module in TBtools (v2.154). The Ka/Ks ratios for duplicated gene events were calculated using KaKs Calculator 2.0 (Zhang, 2022), while Ks density distribution curves were generated with the ggplot2 package in R (Ginestet, 2011).

### 2.5 Transcriptomic analysis

The published sequencing datasets SRR768136, SRR6308182, SRR3744769, SRR7812057, SRR3744786, SRR3744787, SRR3744772, SRR14225142, SRR14225143, SRR14225144, SRR3744766, SRR3744767, SRR3744768, SRR6308190, SRR6308181, SRR6308189, SRR6308188, SRR6308187, SRR6308192, SRR6308191, SRR6308194, SRR6308193, SRR3384922, SRR3384921, SRR3384920, SRR3384919, SRR3384923, SRR3384918, SRR3384917, SRR3384916, SRR3384915, SRR3384914, SRR3384913, SRR3384912, SRR3384911, SRR3384910, SRR7343308, SRR7343309, SRR5405144, SRR6320494, SRR5405142, SRR5405143 and SRR6320572 were downloaded from the Genome Database for Rosaceae (GDR) and used to analyze the expression patterns of *Md2OGD-Hs* in apple seed, young leaf, mature leaf, stem, root, flower, stigmas, styles, ovaries, filaments, anthers, pollen, petals, sepals and receptacles (Jung et al., 2014; Daccord et al., 2017). The transcriptomic analysis information is provided in **Supplementary Table S7**. The expression profiles of *Md2OGD-Hs* under different abiotic stresses, including cold, heat, and salt stress, were analyzed using transcriptome data from previous studies (Li et al., 2021; Mei et al., 2023; Zhang et al., 2024c). Raw sequencing data quality was assessed using FastQC, followed by the retention of the high-quality reads and alignment to the apple reference genome using HISAT2 (v2.2.1) (Andrews, 2010; Kim et al., 2019). Differentially expressed genes (DEGs) were identified through DESeq2 (v1.42.1) implemented in R (v4.3.1), with |log_2_FC| > 1 and adjusted p-value < 0.05 as significance thresholds (Love et al., 2014). The transcriptomic analysis information is provided in **Supplementary Tables S8**–**S12**.

### 2.6 Vector construction and genetic transformation

The coding sequence (CDS) of *MdGA2ox1b* was cloned into the pCambia2300-eGFP vector to construct an overexpression vector (OE), while a 200 bp gene fragment was inserted into the pK7GWIWG2D vector and TRV2 vector to generate an RNA interference (RNAi) construct. The primers used for these cloning procedures are listed in **Supplementary Table S13**. The constructs were then used to transform apple calli or WT plants through *Agrobacterium tumefaciens* (GV3101) as described previously (Li et al., 2023).

### 2.7 Subcellular localization

The *35S*::*MdGA2ox1b-eGFP* construct and the pCambia2300-eGFP empty vector were individually transformed into *A. tumefaciens*. The activated cells were resuspended in an infiltration solution and adjusted to OD_600_ of 0.6 and subsequently injected into *N. benthamiana* leaves for transient transformation. The 35S::AtUGP1-mCherry construct was used as a cytoplasmic marker (Zhang et al., 2023b). Fluorescent protein signals were observed 48–60 h post-injection using a laser scanning microscope (TCS-SP8 SR; Leica, Wetzlar, Germany).

### 2.8 Abiotic stress treatments and physiological measurements

For salt stress, wild-type and transgenic calli with uniform growth status were precisely weighed at 0.1 g and transferred to MS solid medium supplemented with 200 mM NaCl or standard MS medium. Samples were collected after 20 days of dark cultivation at 25 °C for physiological analysis (Wang et al., 2023). For cold stress, calli were inoculated on MS medium in the dark for 25 days at 4°C, with those kept at 25 °C as the control and then harvested and measured (Yang et al., 2023). For heat stress, calli were placed on MS medium with open lids and incubated at 48 °C for 3 h, with those kept at 25 °C as the control. All samples were maintained in the dark before collection, but were collected at different time points for expression pattern analysis. Each stress treatment included 10 culture plates. The fresh weight of apple calli was measured using an analytical balance. The samples were collected, ground into powder using liquid nitrogen, and processed according to the manufacturer’s instructions to determine the activities of superoxide dismutase (SOD), peroxidase (POD), and catalase (CAT). Enzyme activities were measured using detection kits, including the Superoxide Dismutase Activity Assay Kit, Peroxidase Activity Detection Kit, and Catalase Assay Kit (Suzhou Comin Biotechnology Co., Ltd.). Ultraviolet spectrophotometry was performed using a UV-2600 spectrophotometer (Shimadzu Corporation, Tokyo, Japan).

### 2.9 RNA extraction and quantitative reverse transcription PCR analysis

Total RNA was extracted using the Plant Total RNA Extraction Kit (Code No. RE-05014; FOREGENE Biotechnology Co., Ltd., Chengdu, China) following the manufacturer’s instructions. First-strand cDNA was synthesized using the RevertAid First Strand cDNA Synthesis Kit (Code No. K1622; Thermo Fisher Scientific, Waltham, MA, USA) according to the manufacturer’s instructions. The qRT-PCR reaction mixture comprised a 20 μL total volume, including 10 μL Universal Taq SYBR Green qPCR Premix, 0.4 μL each of forward and reverse primers (10 μM), 1 μL cDNA template, and 8.2 μL nuclease-free water. Reactions were carried out on a QuantStudio™ 3 Real-Time PCR System (Thermo Fisher Scientific, Waltham, MA, USA). Relative gene expression levels were calculated using the 2^−ΔΔCt^ method, with *Malate dehydrogenase* (*MdMDH*) and *elongation factor 1-alpha* (*MdEF-1α*) as the internal reference genes, after normalization using the geometric mean of both genes as described previously (Niu et al., 2022). Gene-specific primers (**Supplementary Table S13**) for qRT-PCR were designed using Primer Premier 5 software (Zhai et al., 2008).

### 2.10 Yeast one-hybrid

The promoter sequence of *MdGA2ox1b* was cloned into the pHIS2 vector, and the coding sequence of the transcription factor *MdHY5* was cloned into the pGADT7 vector. The recombinant constructs were then transformed into yeast Y187 competent cells, with the empty pGADT7 vector serving as a control. The transformed yeast cells were plated on SD/-Trp/-Leu dropout medium. Positive clones were subsequently spotted onto SD/-Trp/-Leu/-His selective medium containing 20 mM 3-amino-1, 2, 4-triazole and incubated at 30 °C for 3–5 days to assess yeast growth.

### 2.11 Electrophoretic mobility shift assay

The three bZIP binding elements in the *MdGA2ox1b* promoter were synthesized by Sangon Biotech as biotin-labeled probes and unlabeled competitor probes. Mutant probes with core base mutations were also synthesized as negative controls. The prokaryotically expressed and purified MdHY5-His recombinant protein was incubated with the competitor probes probes at room temperature for 20 min. Unlabeled competitor probes biotin-labeled or mutant probes were then added for competitive binding analysis. After incubation, the reaction products were subjected to non-denaturing polyacrylamide gel electrophoresis, followed by transfer onto a nylon membrane. The DNA-protein complexes were cross-linked by UV irradiation, and biotin signals were detected using a chemiluminescence method.

### 2.12 β-glucuronidase (GUS) assay

The *MdGA2ox1b* promoter-driven GUS reporter vector (*proMdGA2ox1b*::*GUS*) and the 35S promoter-driven effector vector (*35S*::*MdHY5*) or the empty vector were co-transformed into *Agrobacterium tumefaciens* GV3101 and transiently infiltrated into apple calli. The combination of *proMdGA2ox1b*::*GUS* with *35S*::*MdHY5* served as the experimental group, while proMdGA2ox1b:GUS with the empty vector served as the control group. The transformed calli were subjected to various conditions, including control conditions as well as drought, salt, cold, and heat stresses. Following treatment, the samples were immersed in GUS staining solution and incubated at 37 °C in the dark. Meanwhile, GUS activity was quantitatively measured using a fluorometer with 4-methylumbelliferyl-β-D-glucuronide as the substrate. GUS activity was calculated as the fluorescence value of 4-methylumbelliferone produced per unit time per unit protein.

### 2.13 Dual-luciferase assay

The *MdGA2ox1b* promoter was cloned into a firefly luciferase reporter vector (*proMdGA2ox1b*::*LUC*), and the coding sequence of *MdHY5* was cloned into an effector vector containing the 35S promoter (*35S*::*MdHY5*). Th vectors were transformed into *Agrobacterium tumefaciens* GV3101 and transiently infiltrated into apple calli. The combination of *proMdGA2ox1b*::*LUC* with *35S*::*MdHY5* served as the experimental group, while *proMdGA2ox1b*::*LUC* with the empty vector served as the control group. The transformed calli were subjected to normal conditions and various stress treatments. Samples were lysed using a dual-luciferase assay kit, and the activities of firefly luciferase (LUC) and Renilla luciferase (REN) were measured using a luminometer.

### 2.14 Statistical analysis

Statistical analysis was calculated by one-way analysis of variance using SPSS 19.0. Significant differences were analyzed using Tukey’s test, while graphical presentations were performed using GraphPad Prism 10.

## 3 Results

### 3.1 Identification, phylogenetic analysis, motif composition, conserved domains, and gene structure analysis of *Md2OGD-Hs*

To identify the *2OGDs* in apple, we first performed a BLASTP search using Arabidopsis At2OGD protein sequences as queries for preliminary screening. Candidate proteins were further refined through a local HMMER analysis based on the HMM of the conserved 2OG-FeII_Oxy domain (PF03171). After excluding non-2OG-FeII_Oxy-containing members via NCBI Batch CD-Search and SMART, 257 2OGD proteins were identified in apple and designated as Md2OGDs. The predicted *Md2OGDs* ranged from 49 to 690 amino acids, with molecular weights spanning 5.45 to 74.38 kDa and pI between 4.0 and 9.81. Approximately 77.82% of Md2OGDs were in the cytoplasm, 22.96% in the nucleus, 4.28% in the cell membrane and 23.74% in organelles or other compartments (**Supplementary Table S1**). The maximum likelihood phylogenetic tree of the protein sequences of At2OGDs and Md2OGDs constructed using MEGA 12 classified the Md2OGDs into three clades, including DOXA with 15 members, DOXB with 15 members, and DOXC with 227 members (**Supplementary Figure S1**). Within the DOXC clade, each *Md2OGD-H* gene was assigned to a specific subfamily based on its phylogenetic clustering with functionally characterized *2OGD-H* reference genes from Arabidopsis (Swarbreck et al., 2008), tomato (Ding et al., 2020), grape (Gao et al., 2022) and rice (Shu et al., 2007). The nine subfamilies comprise 8 C19-GA2ox, 9 C20-GA2ox, 5 GA20ox, 8 GA3ox, 6 ACO, 6 DAO, 4 LBO, 4 JOX and 8 DMR6/DLO members, totaling 58 *Md2OGD-H* genes, and were named according to their Arabidopsis homologs and chromosomal locations (**Figure 1**; **Supplementary Figure S1**). Therefore, given the pivotal regulatory roles of 2OGD-H proteins in phytohormone signaling transduction and abiotic stress adaptation, 58 *Md2OGD-Hs* of the 257 *Md2OGDs* identified in apple were further characterized in the subsequent functional and mechanistic investigations.

**Figure 1:**
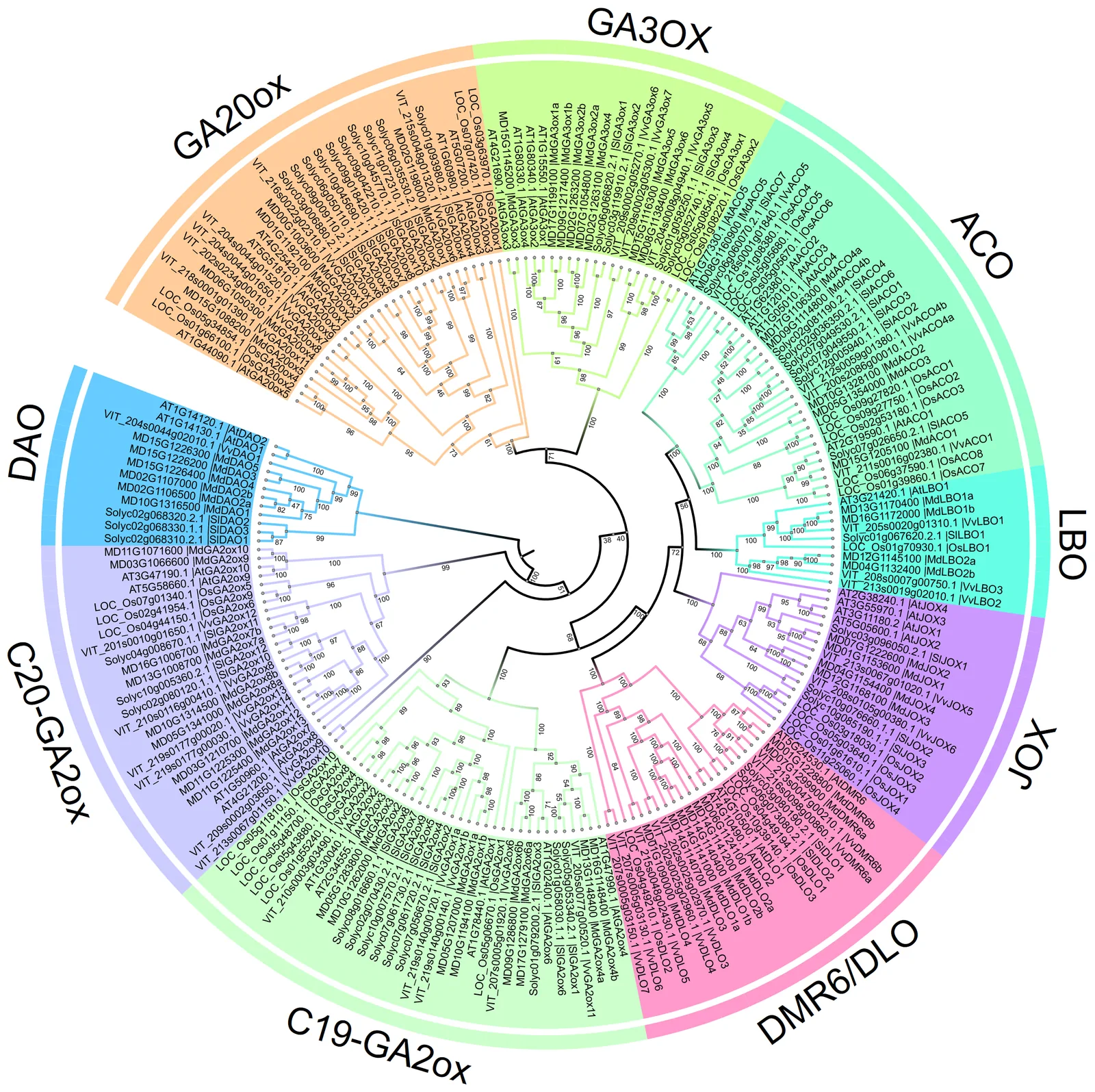
Phylogenetic analysis of Md2OGD-H proteins. The amino acid sequences were aligned using MAFFT and used to construct a phylogenetic tree using MEGA 12 under the maximum likelihood method. Bootstrap values were calculated based on 1000 replicates. The Md2OGD-H proteins were categorised into nine distinct clades, represented by branches with distinct colours.

To further characterize the relationships among Md2OGD-Hs members, we analyzed their motif composition, conserved domains, and gene structures. The domain analysis via Batch CD-Search and SMART showed that 98.28% of Md2OGD-Hs contained the 2OG-FeII_Oxy domain, 91.38% possessed an N-terminal DIOX_N domain, and 89.66% co-harboured both domains, underscoring their conserved catalytic roles (**Supplementary Figure S2**; **Supplementary Table S2**). The MEME-based motif prediction identified 20 conserved motifs with 8–50 amino acids in clade-specific distributions. Motifs 13 and 15 were exclusive to MdC19-GA2oxs, motif 8 was unique to MdGA3oxs, MdDAOs universally retained motif 14, MdLBOs and MdJOXs shared motif 11, MdDMR6/DLOs harboured motifs 6, 17, and 18, while no clade-specific motifs were detected in MdC20-GA2oxs, MdGA20oxs or MdACOs (**Supplementary Figure S2**; **Supplementary Table S3**). Gene structure analysis showed 1 to 4 exons, with 15 genes lacking introns, 10 genes containing one intron, 30 genes harbouring two introns, and only three genes exhibiting > 3 introns (**Supplementary Figure S2**). These findings collectively highlight the evolutionary and functional divergence within the *Md2OGD-Hs* subfamily.

### 3.2 Analysis of cis-acting elements in *Md2OGD-Hs*

The cis-acting elements are the key players in transcriptional regulation and are widely involved in plant photomorphogenesis, phytohormone responses, stress adaptation, and plant growth and development. To investigate the upstream regulatory mechanism of the *Md2OGD-Hs* gene, the cis-acting elements in the promoter regions (2, 000 bp upstream of the ATG start codon) of the 58 *Md2OGD-H* genes were annotated using the PlantCARE database. Twenty-three types of cis-acting elements were identified, including eight types associated with light signal responses, eight types belonging to hormone responses, four types related to stress adaptation and three types correlated with growth and development (**Supplementary Table S4**). All the *Md2OGD-H* gene promoters except for *MdGA3ox1b* and *MdGA3ox5* contained cis-acting elements related to light signal responses, phytohormone responses, and stress adaptation. Light-responsive cis-acting elements were the most abundant, with most elements exhibiting high-frequency repetition (**Figure 2**). The distribution patterns of these cis-elements showed that ABRE was enriched in the GA2ox subfamily, where 15 of 17 members contained this motif at a proportion of 88.2%, and in the DMR6/DLO subfamily, where 7 of 8 members contained this motif at 87.5%. In contrast, G-box motifs were present in almost all Md2OGD-H promoters, without obvious subfamily-specific enrichment. These enrichment trends provide predictive clues for potential regulatory networks. Approximately 62.07% of the *Md2OGD-H* promoters harboured elements associated with growth and development, suggesting their potential role in regulating hormone biosynthesis and metabolism to influence plant growth. Notably, G-box and ABA-responsive element (ABRE) showed similar distribution patterns in the promoters of certain *Md2OGD-H* members, suggesting that bZIP-binding motifs are present in these promoters, providing candidate targets for future regulatory studies.

**Figure 2:**
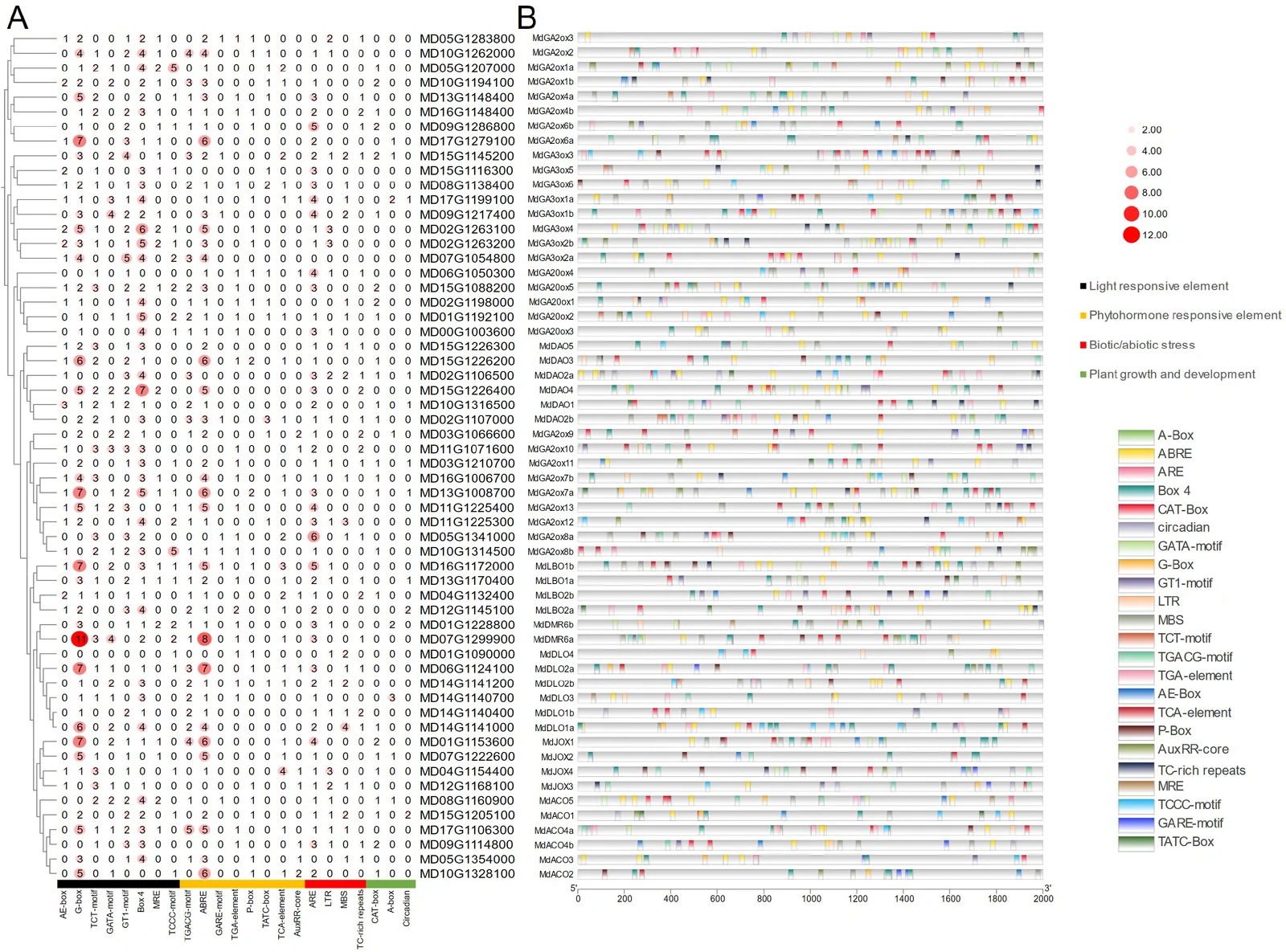
Analysis of cis-acting elements in the promoter regions of *Md2OGD-Hs* based on the PlantCARE database. **(A)** Bubble heatmap displays of the distribution of different cis-acting elements across each *Md2OGD-H* gene. The numbers indicate the count of cis-acting elements. **(B)** The element position map illustrates the locations of cis-acting elements, with colours denoting their functional categories. The elements related to light signal response (black), phytohormone response (orange), stress adaptation (red), and plant growth and development (green) are highlighted.

### 3.3 Chromosomal distribution and gene duplication of *Md2OGD-Hs*

The evolutionary dynamics of the *Md2OGD-Hs* gene family in apple were investigated through chromosomal distribution, synteny analysis, and synonymous substitution rate (Ks) profiling. Apart from the *MdGA20ox3*, which was not found in any chromosome, a striking chromosomal bias was observed among the 57 *Md2OGD-H* genes. Chr15 harboured seven genes, followed by Chr02 and Chr10 with five genes each, while Chr03, Chr04, Chr06, Chr08, and Chr12 contained only two genes each (**Figure 3A**).

**Figure 3:**
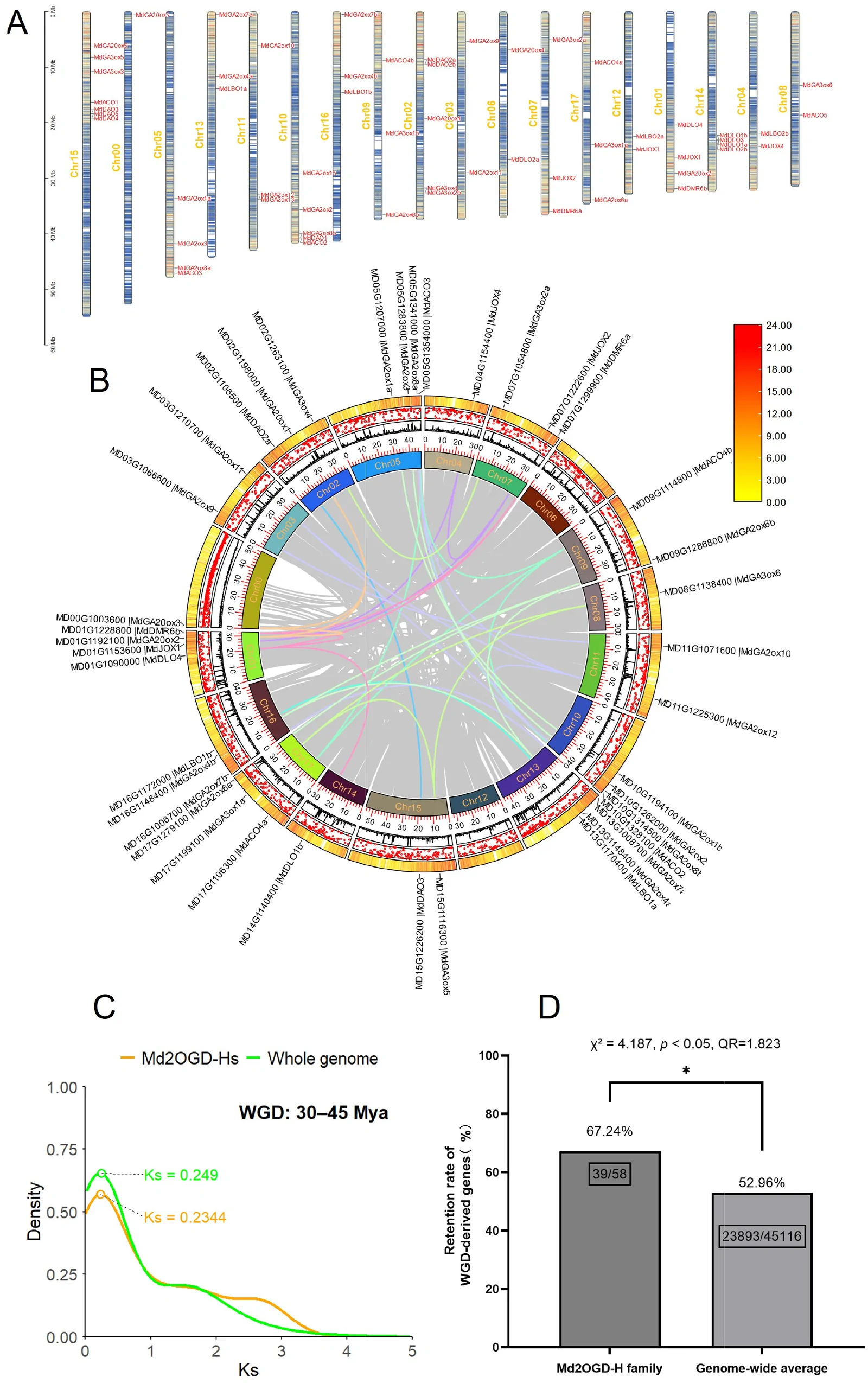
Chromosomal distribution and gene duplication of *Md2OGD-Hs*. **(A)** Chromosomal positions of *Md2OGD-Hs* genes across 17 apple chromosomes. A scale bar indicates a chromosomal distance of 10.0 Mb. **(B)** Syntenic analysis of *Md2OGD-Hs* genes on apple chromosomes. Chromosomes are randomly coloured and labelled with their respective names. Colour-coded lines represent segmental duplication events within different subfamilies. **(C)** Ks value distribution of *Md2OGD-Hs* segmental duplication pairs compared to the apple whole-genome background. **(D)** Comparison of the proportion of WGD-derived genes in the Md2OGD-H family with that in the apple genome-wide background. *P < 0.05. *, indicates a statistically significant difference between the proportion of WGD-derived genes in the Md2OGD-H family (67.24%) and the genome-wide average (52.96%), as determined by the chi-square test (*P < 0.05; P = 0.041).

To further explore the potential relationships and evolutionary trends of segmental and tandem duplication events in *Md2OGD-H* genes, we examined their collinear relationships across apple chromosomes. Thirty-four pairs of segmental duplication events were identified, including three pairs in *MdGA20oxs*, four pairs in *MdGA3oxs*, four pairs in *MdDMR6/DLOs*, six pairs in *MdC19-GA2oxs*, seven pairs in *MdC20-GA2oxs*, five pairs in *MdACOs*, three pairs in *MdJOXs*, and one pair each in *MdLBOs* and *MdDAOs*. (**Figure 3B**; **Supplementary Table S5**). The Ks peak for the 34 segmental duplication pairs within the *Md2OGD-H* family was 0.2344, closely matching the apple whole-genome Ks peak at 0.249 (**Figure 3C**). This Ks value corresponds to the apple lineage-specific WGD event dated to 30–45 million years ago (Velasco et al., 2010; Daccord et al., 2017). In addition, 39 of the 58 *Md2OGD-H* genes were located within WGD-derived collinear blocks, accounting for 67.24% of the family, which is significantly higher than the genome-wide average of 52.96% (χ² = 4.187, *p* = 0.041, OR = 1.823) (**Figure 3D**; **Supplementary Table S5**). Furthermore, the Ka/Ks ratios for all 34 duplication pairs ranged from 0.12 to 0.46, all far below 1, indicating purifying selection (**Supplementary Table S5**). Collectively, these data support that the expansion of *Md2OGD-Hs* primarily occurred during the WGD period in apple.

To further investigate the evolutionary relationships of *2OGD-H* genes among different plant species, we performed interspecies chromosomal collinearity analysis with genes from apple, Arabidopsis, tomato, grape, and rice. The *Md2OGD-Hs* formed 44 collinear gene pairs with *At2OGD-Hs*, 53 pairs with *Sl2OGD-Hs*, 56 pairs with *Vv2OGD-Hs* and 17 collinear gene pairs with *Os2OGD-Hs*, indicating a closer evolutionary relationship of *Md2OGD-Hs* with *Vv2OGD-Hs* and a distant relationship with *Os2OGD-Hs* (**Figure 4**; **Supplementary Table S6**). Every one of the 170 pairs belongs to the same functional subfamily, with no cross-subfamily pairings detected. To further assess whether pairings within the same functional subfamily imply functional conservation, we compiled experimental evidence from multiple species for each subfamily. GA2ox has been confirmed to inactivate gibberellins in both Arabidopsis and apple (Yan et al., 2024); GA20ox and GA3ox have been verified to function in gibberellin biosynthesis in Arabidopsis, rice, and tomato (Rieu et al., 2008; Itoh et al., 2001); ACO has been demonstrated to catalyze ethylene synthesis in tomato and apple (Alexander and Grierson, 2002; Jia et al., 2022); DAO has been shown to oxidatively inactivate auxin in rice and Arabidopsis (Zhao et al., 2013; Zhang et al., 2016); JOX has been validated to hydroxylate and inactivate jasmonic acid in Arabidopsis and tobacco (Caarls et al., 2017; Tang et al., 2020); and DMR6/DLO has been confirmed to degrade salicylic acid in Arabidopsis and tomato (Zhang et al., 2017; Thomazella et al., 2021). For each of these subfamilies, independent transgenic functional assays in multiple species have demonstrated that their biochemical functions are conserved across the plant kingdom.

**Figure 4:**
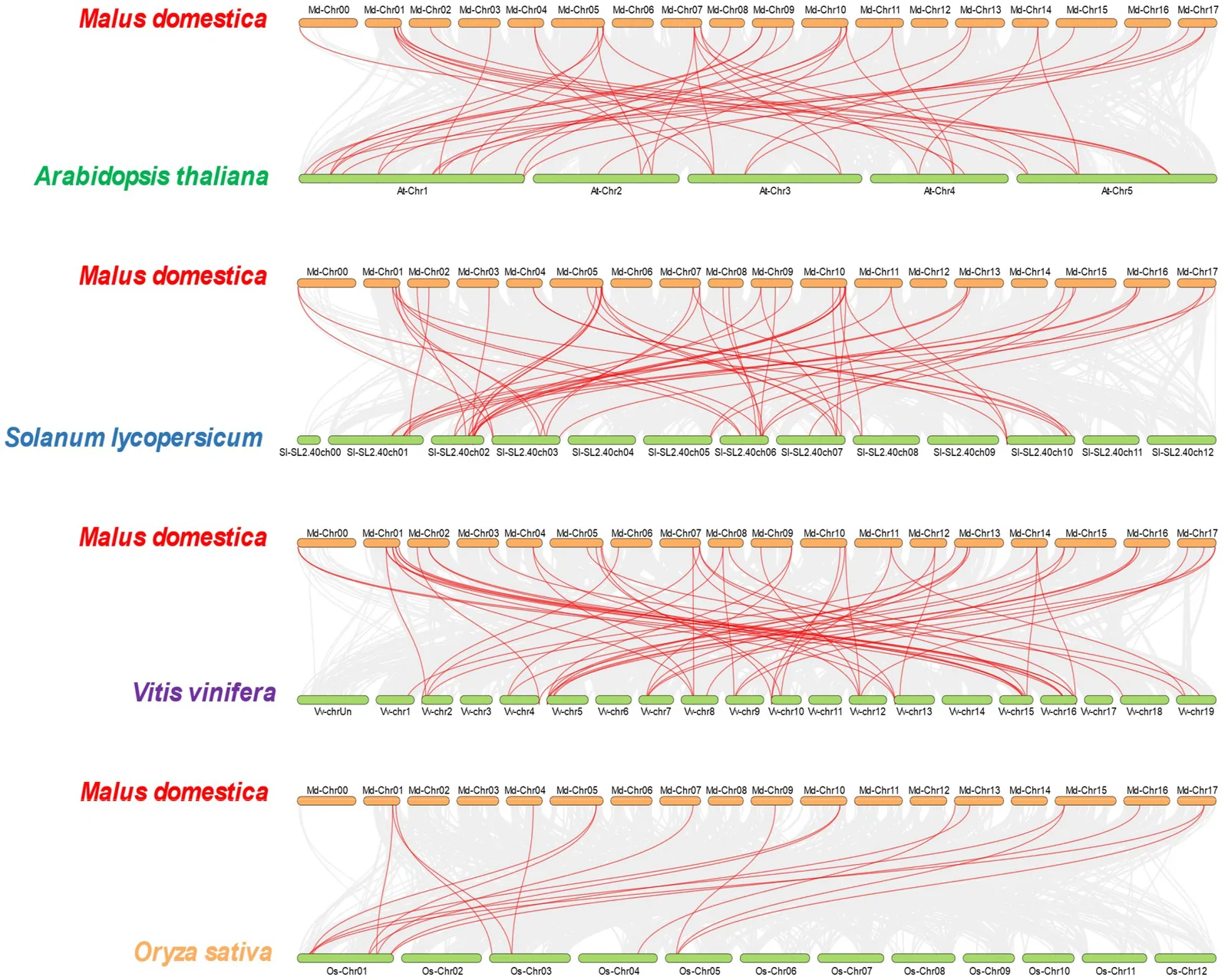
Interspecies chromosomal collinearity analysis of *2OGD-H* genes in apple, Arabidopsis, tomato, grape, and rice. Grey lines indicate intra-genomic collinear blocks, while the red lines represent collinear gene pairs.

### 3.4 Tissue-specific expression pattern analysis of *Md2OGD-Hs*

We obtained tissue-specific expression profiles of *Md2OGD-H* genes under normal developmental conditions from published datasets. The results showed that 39, 30, 32, 25, 31, 38, 23, 28, 28, 33, 29, 7, 26, 28, and 29 *Md2OGD-Hs* genes were expressed (FPKM > 0.5) in seed, young leaf, mature leaf, stem, root, flower, stigmas, styles, ovaries, filaments, anthers, pollen, petals, sepals and receptacles, respectively (**Figure 5A**; **Supplementary Table S7**). Interestingly, *MdGA2ox1b*, *MdDAO2a*, *MdDAO2b*, *MdJOX4*, *MdACO3*, *MdACO4a* and *MdACO4b* displayed high expression levels (FPKM > 5) in all tissues except in pollen, suggesting their potential involvement in apple development across multiple tissues. Furthermore, analysis of expression patterns during distinct fruit developmental stages revealed that 26 genes, including *MdGA2ox1a*, *MdGA2ox1b*, *MdGA3ox3*, *MdGA3ox2a*, *MdDAO1*, *MdDAO2a*, *MdDAO2b*, *MdDAO3*, *MdDAO4*, *MdGA2ox7b*, *MdGA2ox9*, *MdGA2ox10*, *MdGA2ox11*, *MdLBO1a*, *MdLBO2b*, *MdDMR6a*, *MdDMR6b*, *MdDLO2a*, *MdJOX1*, *MdJOX2*, *MdJOX3*, *MdJOX4*, *MdACO1*, *MdACO3*, *MdACO4a* and *MdACO4b*, exhibited dynamic expression changes that correlated with fruit ripening (**Figure 5B**; **Supplementary Table S7**). This implies the regulatory roles of these genes in apple fruit maturation, possibly through hormone metabolism or signaling pathways. Therefore, the *Md2OGD-Hs* gene family likely plays critical roles in tissue development and fruit ripening in apple.

**Figure 5:**
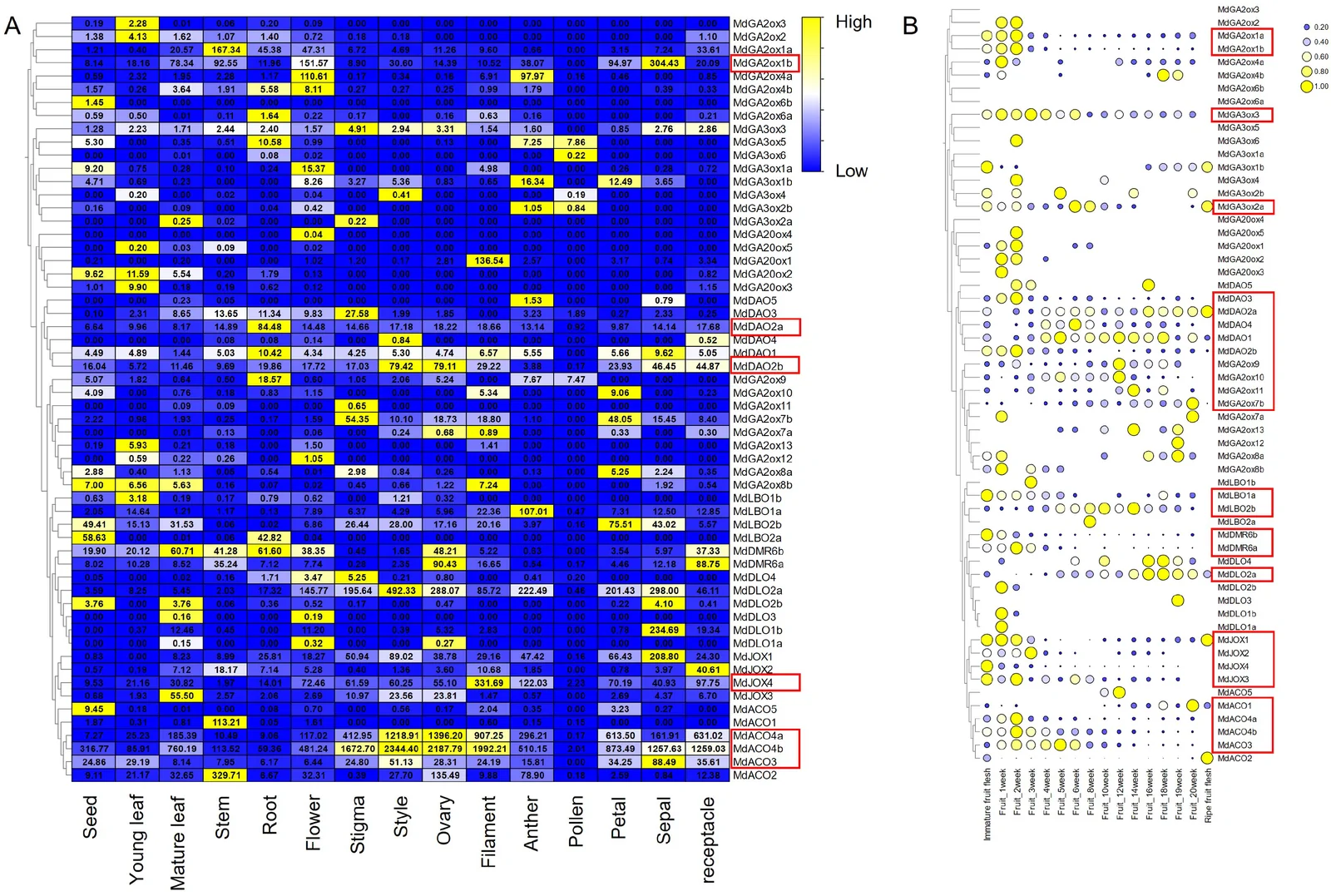
Expression profile analysis of *Md2OGD-Hs* in different tissues and fruit developmental stages based on RNA-sequencing. **(A)** Expression profiles of *Md2OGD-Hs* in different apple tissues. Red rectangles highlight seven genes exhibiting high expression across all tissues except in pollen. **(B)** Expression patterns of each *Md2OGD-Hs* during different fruit developmental stages. Red rectangles show 26 genes potentially associated with fruit ripening.

### 3.5 Expression patterns of *Md2OGD-Hs* under abiotic stress

By integrating both in-house established and publicly accessible transcriptome datasets of apple under salt, drought, cold and heat stresses, we conducted a systematic analysis of the expression dynamics of Md2OGD-H members across distinct stress regimes. Eight *Md2OGD-H* genes were differentially expressed under salt stress, with three, including *MdGA2ox1b*, *MdDLO1a*, and *MdJOX4* genes upregulated, and five, including *MdGA2ox1a*, *MdGA3ox1a*, *MdGA20ox2*, *MdDAO3* and *MdLBO2b* genes downregulated (**Figure 6A**; **Supplementary Table S8**). Thirteen *Md2OGD-H* genes exhibited differential expression during drought stress, including *MdGA2ox7b*, *MdGA2ox8a*, and *MdGA2ox8b* which were upregulated, and *MdGA2ox1b*, *MdGA3ox1a*, *MdGA20ox2*, *MdGA2ox9*, *MdDMR6b*, *MdDMR6a*, *MdJOX3*, *MdACO4a*, *MdACO4b*, and *MdACO3* genes which were downregulated (**Figure 6B**; **Supplementary Table S9**). Among the 12 *Md2OGD-H* genes, only *MdDAO1* was downregulated under low-temperature stress, while 11 genes, including *MdGA2ox1a*, *MdGA2ox1b*, *MdGA2ox4a*, *MdGA2ox4b*, *MdGA20ox3*, *MdGA2ox7a*, *MdDMR6b*, *MdDLO1b*, *MdDLO1a*, *MdJOX1*, and *MdACO4a* were upregulated, indicating a significant role of *Md2OGD-Hs* in apple cold stress adaptation (**Figure 6C**; **Supplementary Table S10**). Seventeen were potentially involved in responses. Six *Md2OGD-H* genes, including *MdGA2ox1b*, *MdGA2ox7b*, *MdDMR6b*, *MdJOX1*, *MdJOX2* and *MdJOX4* were upregulated by high-temperature stress, while eleven *Md2OGD-H* genes, including *MdGA3ox1a*, *MdGA20ox3*, *MdDAO1*, *MdDAO2b*, *MdGA2ox8b*, *MdLBO1a*, *MdLBO2b*, *MdDMR6a*, *MdDLO2b*, *MdJOX3*, and *MdACO3* genes were downregulated (**Figure 6D**; **Supplementary Table S11**).

**Figure 6:**
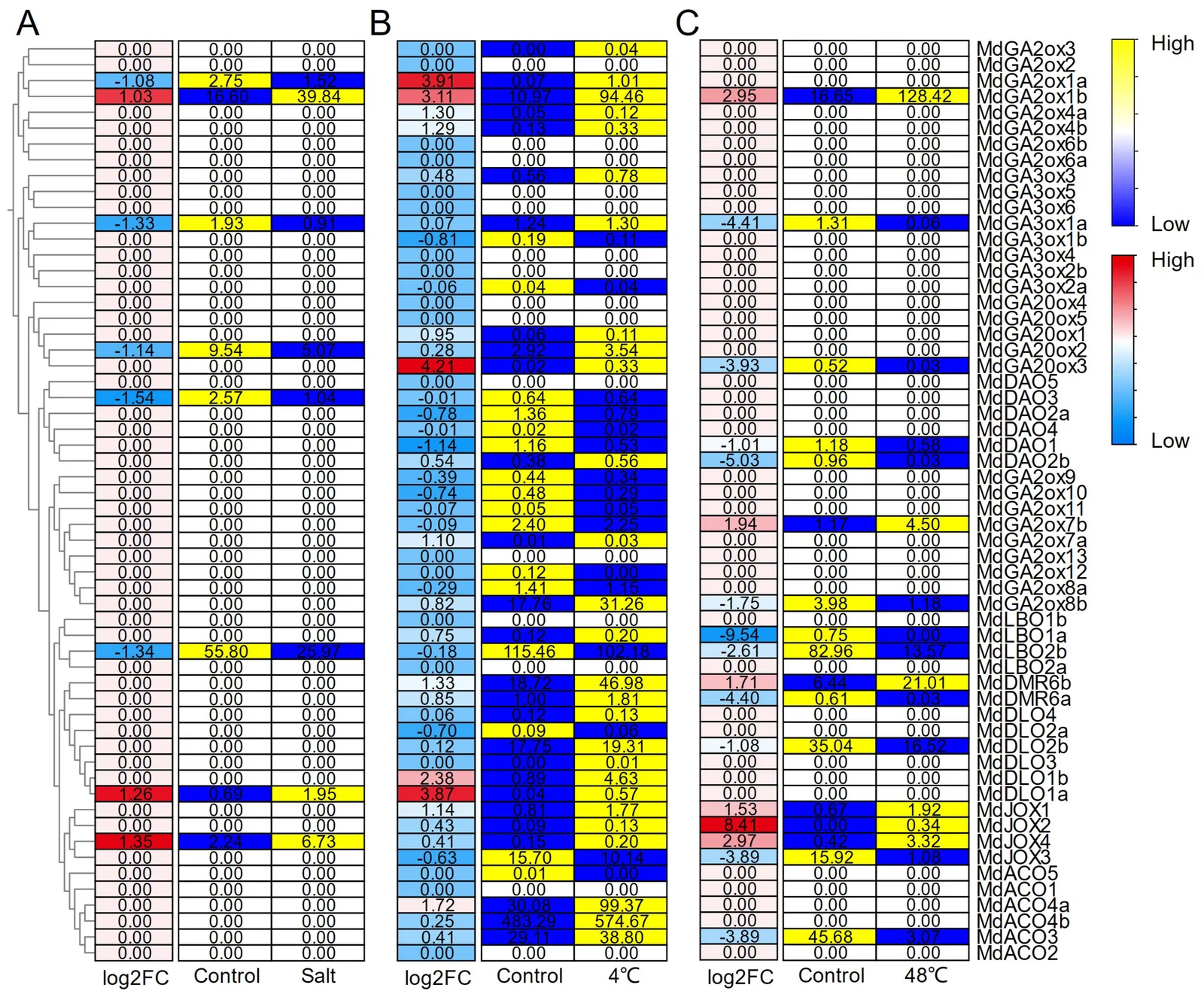
Expression profiling of *Md2OGD-Hs* under different stresses in apple based on RNA-sequencing. Expression patterns of *Md2OGD-Hs* under salt stress **(A)**, low-temperature stress **(B)**, and high-temperature stress **(C)**. In the heatmap, yellow and red indicate high expression levels, while blue represents low expression. The threshold for defining differentially expressed genes was set as |log2FC| > 1.

Among the DEGs, *MdGA2ox1b* was differentially expressed under all four stress conditions (**Figure 7A**; **Supplementary Table S12**), suggesting its potential central role in apple abiotic stress responses. Therefore, we validated its dynamic expression pattern in apple calli under salt, drought, cold and heat stresses using qRT-PCR. The results demonstrated that *MdGA2ox1b* rapidly responded to salt stress, peaking at 12 h with a 4.9-fold change, cold stress peaking at 12 h with a 10.59-fold induction, and heat stress peaking at 0.25 h with 10.05-fold induction. (**Figure 7B**).

**Figure 7:**
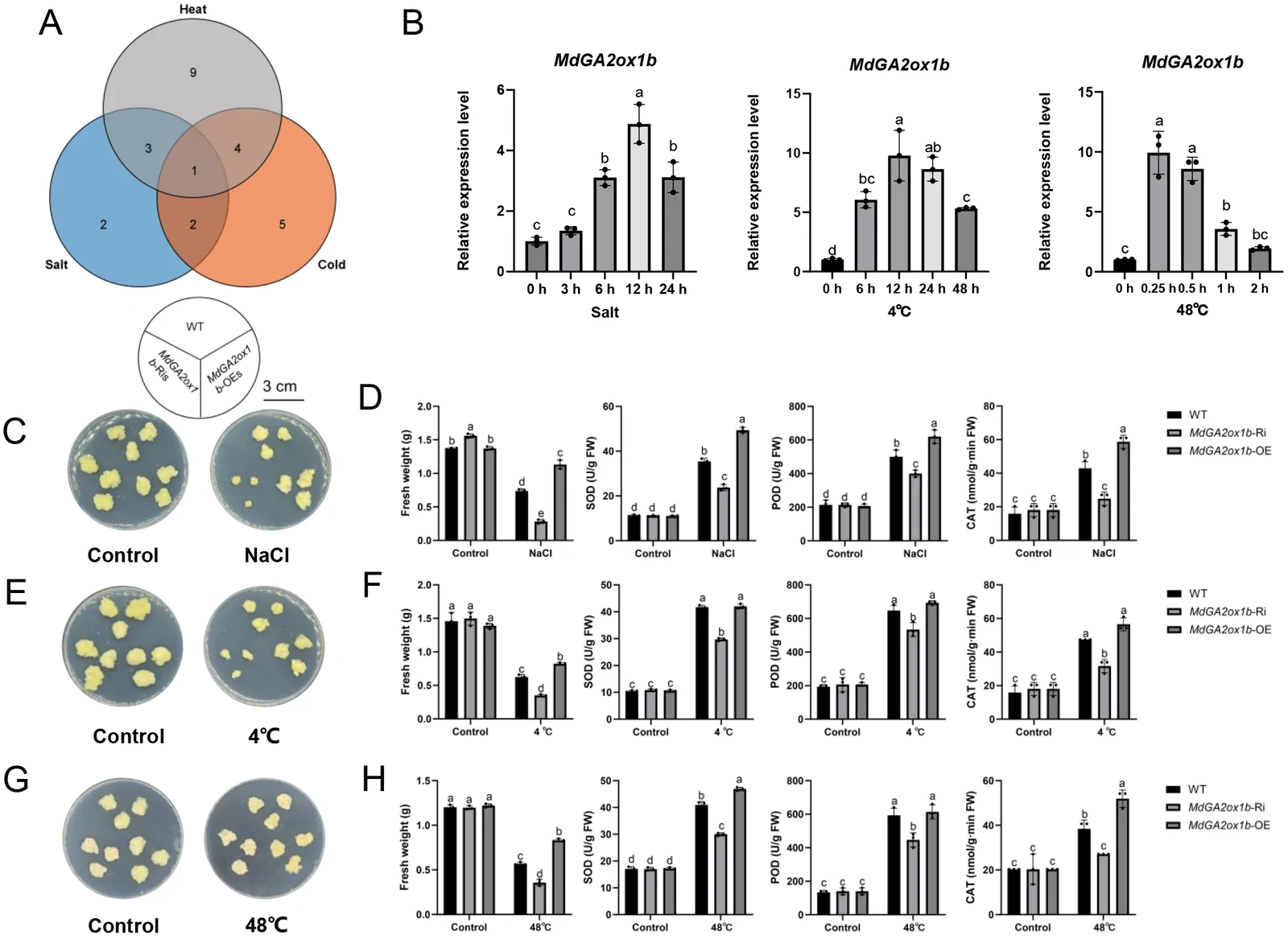
Identification and functional characterization of *MdGA2ox1b* in abiotic stress of apple calli. **(A)** Venn diagram showing the MdGA2ox1b gene as a common differentially expressed gene across four stresses based on RNA-seq analysis (threshold: |log2FC| > 1). **(B)** The RT-qPCR analysis of *MdGA2ox1b* expression under salt, drought, cold and heat stresses. Phenotypic comparison, fresh weight and antioxidant enzyme activities of wild-type (WT) and *MdGA2ox1b* transgenic apple calli under salt stress **(C, D)**, drought stress **(E, F)**, cold stress **(G, H)** and heat stress **(I, J)**. Letters indicate statistically homogeneous groups following a one-way analysis of variance with Tukey’s *post hoc* test, with *P* < 0.05 as the significance level.

### 3.6 *MdGA2ox1b* positively regulates salt, cold, and heat tolerance in apple calli

Based on the transcriptomic and qRT-PCR results, further functional analysis of the stress-responsive candidate gene *MdGA2ox1b* was conducted. Subcellular localization using confocal microscopy showed that MdGA2ox1b-eGFP fluorescence was detected in both the cytoplasm and nucleus. Co-localization with the cytoplasmic marker 35S::AtUGP1-mCherry confirmed its cytoplasmic distribution, and nuclear fluorescence was also clearly observed. These results are consistent with the Plant-mPLoc prediction, which also indicated cytoplasmic and nuclear localization (**Supplementary Figure S3A**; **Supplementary Table S1**). We also examined the relative expression levels of *MdGA2ox1b* across various apple roots, stems, leaves, flowers and mature fruits. Hence, *MdGA2ox1b* exhibited the lowest expression in roots, while its expression in flowers was remarkably upregulated, reaching 10.41-fold higher than that in roots (**Supplementary Figure S3B**).

To determine the role of *MdGA2ox1b* gene in response to abiotic stress in apples, we obtained transgenic apple calli with overexpressed (OE) and silenced (Ri) *MdGA2ox1b* (**Supplementary Figure S4**), and systematically evaluated their physiological responses to salt, cold, and heat stresses. For all three stress treatments, statistical comparisons of the control samples showed no significant differences in SOD, POD, or CAT activities among WT, OE, and Ri lines under their respective non-stressed conditions. All the calli exposed to 200 mM NaCl showed inhibited growth with significant differences, while the Ri calli were dehydrated and brown by the 20^th^ day of cultivation and had significantly decreased fresh weight by 61.54% compared to wild-type (WT). However, OE calli grew significantly better than WT, with fresh weight loss (17.72%) lower than WT (46.36%). The activity of SOD, POD, and CAT in OE calli was 1.4, 1.24, and 1.37-fold higher than those in WT, respectively, while Ri calli showed significantly reduced enzyme activities, suggesting that *MdGA2ox1b* may alleviate salt stress by enhancing reactive oxygen species (ROS) scavenging capacity (**Figures 7C, D**). After 25 days of exposure to 4 °C, the OE calli exhibited significantly higher cold tolerance, maintaining normal growth status with fresh weight approximately 31.37% higher than WT, while Ri calli suffered severe growth inhibition, with their fresh weights reduced by 76.62%. Though the activities of SOD, POD, and CAT in OE calli were not significantly different from those of WT, the Ri calli exhibited only 71.18, 82.47 and 66.67% of WT levels, respectively (**Figures 7E, F**). Under heat stress, OE apple calli were pale yellow and compact, with their fresh weight reduced by 60.23% of WT, while WT and Ri calli appeared bleached and loose, with Ri calli showing more severe damage. The activities of SOD and CAT in OE calli were significantly higher than those in WT, while Ri calli displayed 26.84, 24.92 and 29.41% reductions in SOD, POD, and CAT activities compared to WT, respectively (**Figures 7G, H**). This demonstrates that *MdGA2ox1b* may enhance heat tolerance by selectively activating specific antioxidant enzyme pathways.

### 3.7 MdHY5 binds to the promoter of MdGA2ox1b and activates its expression

To investigate which transcription factors regulate the expression of *MdGA2ox1b*, we performed yeast one-hybrid (Y1H) assay and identified a bZIP transcription factor, MdHY5. The Y1H assay showed that yeast cells co-transformed with *MdHY5*-pGADT7 and *proMdGA2ox1b*-pHIS2 grew normally on SD/-Trp/-Leu/-His medium containing 20 mM 3-AT, whereas those co-transformed with pGADT7 and *proMdGA2ox1b*-pHIS2 failed to grow (**Figure 8A**). Further analysis revealed that the *MdGA2ox1b* promoter contains three bZIP-binding motifs, designated P1, P2, and P3. EMSA results showed that the MdHY5-His recombinant protein bound all three motifs *in vitro*, and competition assays indicated that this binding affinity decreased with increasing concentrations of unlabeled competitor probes. Moreover, MdHY5-His did not bind probes with mutations in the core binding sites (**Figure 8B**). To explore the regulatory relationship between MdHY5 and *MdGA2ox1b*, we performed GUS assays. The results showed that under various stress conditions, apple calli co-expressing *35S*::*MdHY5* and *proMdGA2ox1b*::*GUS* exhibited deeper GUS staining and significantly higher GUS activity compared with those co-expressing the empty vector and *proMdGA2ox1b*::*GUS*, with increases of 1.69, 6.19, 5.15, 13.55, 6.38-fold under control, drought, salt, cold, and heat stress, respectively (**Figure 8C**). Dual-luciferase assays yielded similar results, showing that luciferase activity was significantly higher in calli co-expressing *35S*::*MdHY5* and *proMdGA2ox1b*::*LUC* than in the control under different stress conditions, with increases of 2.52, 5.39, 5.52, 13.11, 6.38-fold under control, drought, salt, cold, and heat stress, respectively (**Figure 8D**). Collectively, these results demonstrate that MdHY5 directly binds to the promoter of *MdGA2ox1b* and activates its transcription, and that this activation is further enhanced under multiple stress conditions, with the most pronounced enhancement observed under cold stress.

**Figure 8:**
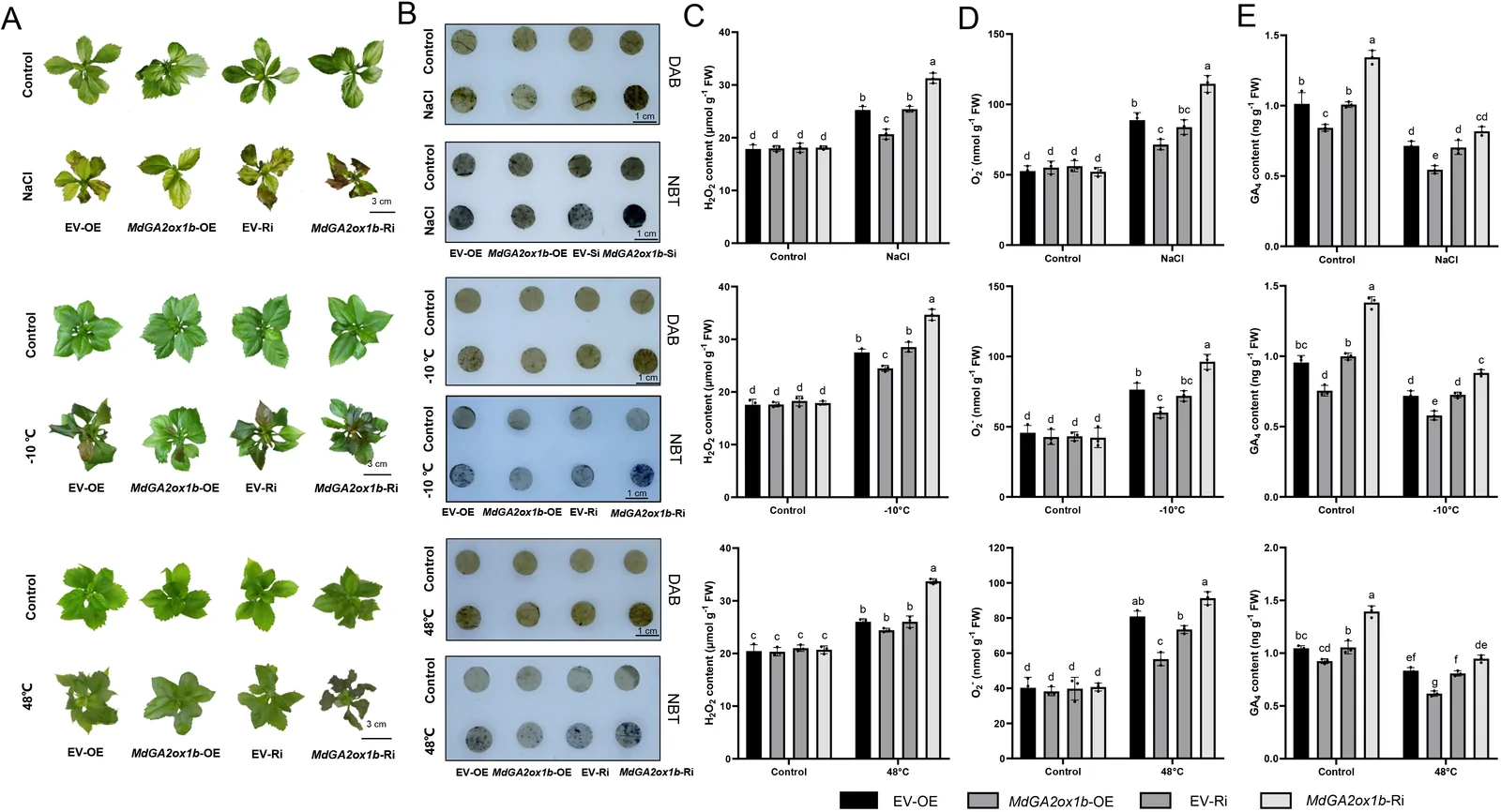
MdHY5 binds to the promoter of *MdGA2ox1b* and activates its expression. **(A)** Yeast one-hybrid assay. 3-AT, 3-amino-1, 2, 4-triazole. **(B)** *MdGA2ox1b* promoter component and electrophoretic mobility shift assay. **(C)** Competitive electrophoretic mobility shift assay. Mut, probes in which the 5′-CACGTG-3′ motifs were replaced by 5′-AAAAAA-3′. **(D)** GUS assay under different stress conditions. Bar = 2 cm. E1 and E2 represent the combination of Effector1 + Reporter and Effector2 + Reporter, respectively. **(E)** Dual luciferase assay under different stress conditions. LUC, firefly luciferase; REN, renilla luciferase. Bar = 2 cm. E1 and E2 represent the combination of Effector1+Reporter and Effector2+Reporter, respectively. Data are means ± SD (n=3).

## 4 Discussion

The *2OGD* family is essential for plant hormone biosynthesis and abiotic stress responses, and its molecular basis of functional diversification has been a research focus in plant evolution and stress adaptation (Kawai et al., 2014). In this study, we identified 257 *Md2OGD* genes in apple, including 11 DOXA, 15 DOXB, and 227 DOXC members. Within DOXC, 58 *Md2OGD-Hs* associated with hormone biosynthesis and metabolism were further characterized (**Figure 1**; **S1**). Their expansion aligns with observations in Arabidopsis, tomato, and grape (Swarbreck et al., 2008; Ding et al., 2020; Gao et al., 2022), reflecting potential conserved roles in hormonal regulation. The 2OG-FeII_Oxy and DIOX_N domains were highly conserved in *Md2OGD-Hs* (**Supplementary Figure S2**; **Supplementary Table S2**), supporting their indispensable roles in catalytic oxidation (Kawai et al., 2014). The 2OG-FeII_Oxy domain, characterized by a conserved His/Asp-Xaa-His triad, coordinates Fe(II) binding and oxygen activation during the decarboxylation of 2OG. The DIOX_N conserved domain, which is localized at the N-terminus, likely enhances the polarity and solvent miscibility of *Md2OGD-Hs*, concurrently conferring oxidative reactivity that facilitates rapid environmental adaptation in apple (Wang et al., 2024). Additionally, motif composition analysis demonstrated that all clades contained conserved motifs except the MdC20-GA2oxs, MdGA20oxs, and MdACOs subgroups, which lacked lineage-specific motifs compared to other branches (**Supplementary Figure S2**; **Supplementary Table S3**). The clade-restricted distribution patterns of these motifs exhibited phylogenetic collinearity with functional divergence, suggesting that these structural domains may act as catalytic signatures involved in substrate selection or enzymatic activity modification (Jinkerson et al., 2024; Goldenberg et al., 2025). The gene structure analysis revealed significant heterogeneity among family members, with exons ranging from 1 to 4, and intron architectures exhibiting polymorphic distributions (**Supplementary Figure S2**). This stepwise gradient in intron numbers suggests divergent structural evolutionary trajectories (Roy and Gilbert, 2006).

Cis-element analysis identified light-responsive (G-box) and stress-related (ABRE) motifs enriched in the promoters of *Md2OGD-Hs* (**Figure 2**; **Supplementary Table S4**). G-box motifs were detected in 48 Md2OGD-H promoters, consistent with the known function of G-box as a light-responsive element in other species (Liu et al., 2019a; Liu et al., 2019b), while the ABRE motifs, which are recognized by bZIP transcription factors such as AREB/ABFs, were found in 50 members, consistent with the known association of ABRE with stress-responsive gene regulation. For instance, the SlAREB1/SlABI3-1/SlABI5 module, induced by abscisic acid (ABA), regulates the expression of *SlDAO2*, indicating how ABA may modulate indole acetic acid (IAA) homeostasis to inhibit tomato hypocotyl elongation (Liu et al., 2023). *MdGA2ox1b*, uniquely induced under salt, cold, and heat stresses (**Figures 6**, **7**), harboured clustered ABRE/G-box elements, raising the possibility of light or ABA signaling involvement, which remains to be experimentally tested. Consistent with this, previous research has shown that the Arabidopsis bZIP transcription factor AtHY5 binds to the G-box in the promoter of *AtGA2ox7*, where it integrates light and gibberellin signals to regulate hypocotyl elongation (Xiong et al., 2023). The cis-element analysis described above provides predictive insights into potential regulatory networks. These predictions are distinct from experimentally validated interactions. In this study, we experimentally validated one such predicted interaction, namely MdHY5 binding to the MdGA2ox1b promoter and activating its transcription as shown in **Figure 8**, while other predicted regulatory relationships await future investigation.

**Figure 9:**
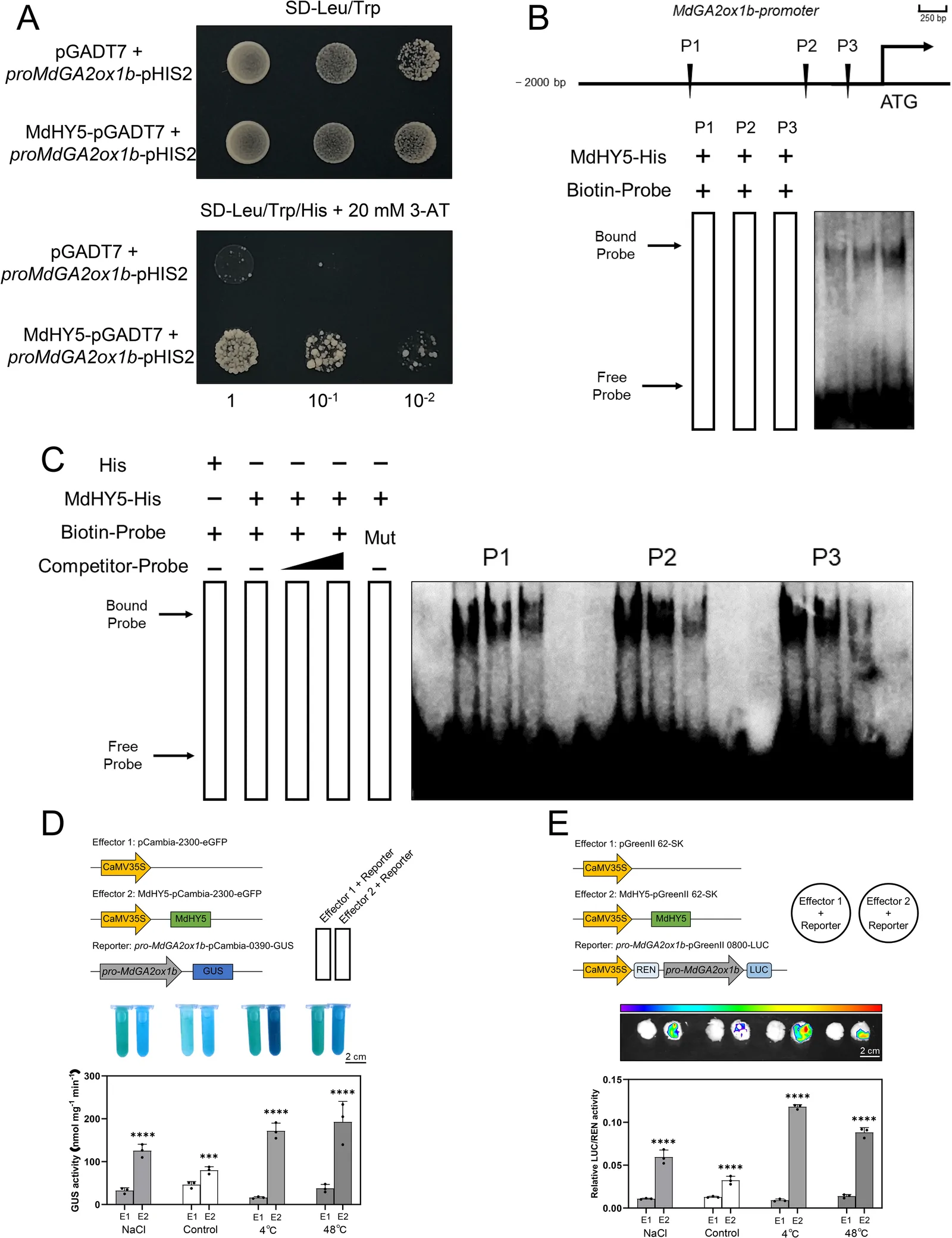
MdHY5 binds to the MdGA2ox1b promoter and activates its transcription. **(A)** Yeast one-hybrid assay showing the interaction between MdHY5 and the MdGA2ox1b promoter. 3-AT, 3-amino-1,2,4-triazole. **(B)** Schematic diagram of the MdGA2ox1b promoter containing three probe regions (P1, P2, and P3) and electrophoretic mobility shift assay (EMSA) analysis. **(C)** Competitive EMSA showing the specific binding of MdHY5 to the MdGA2ox1b promoter using biotin-labeled, competitor, and mutant probes. **(D)** GUS reporter assay showing the transcriptional activation of the MdGA2ox1b promoter by MdHY5 under different conditions. **(E)** Dual-luciferase reporter assay showing the effect of MdHY5 on MdGA2ox1b promoter activity under different conditions. LUC, firefly luciferase; REN, Renilla luciferase. Data are presented as means ± SD (n = 3). The asterisks indicate significant differences at different levels (***P < 0.001 and ****P < 0.0001).

**Figure 10:**
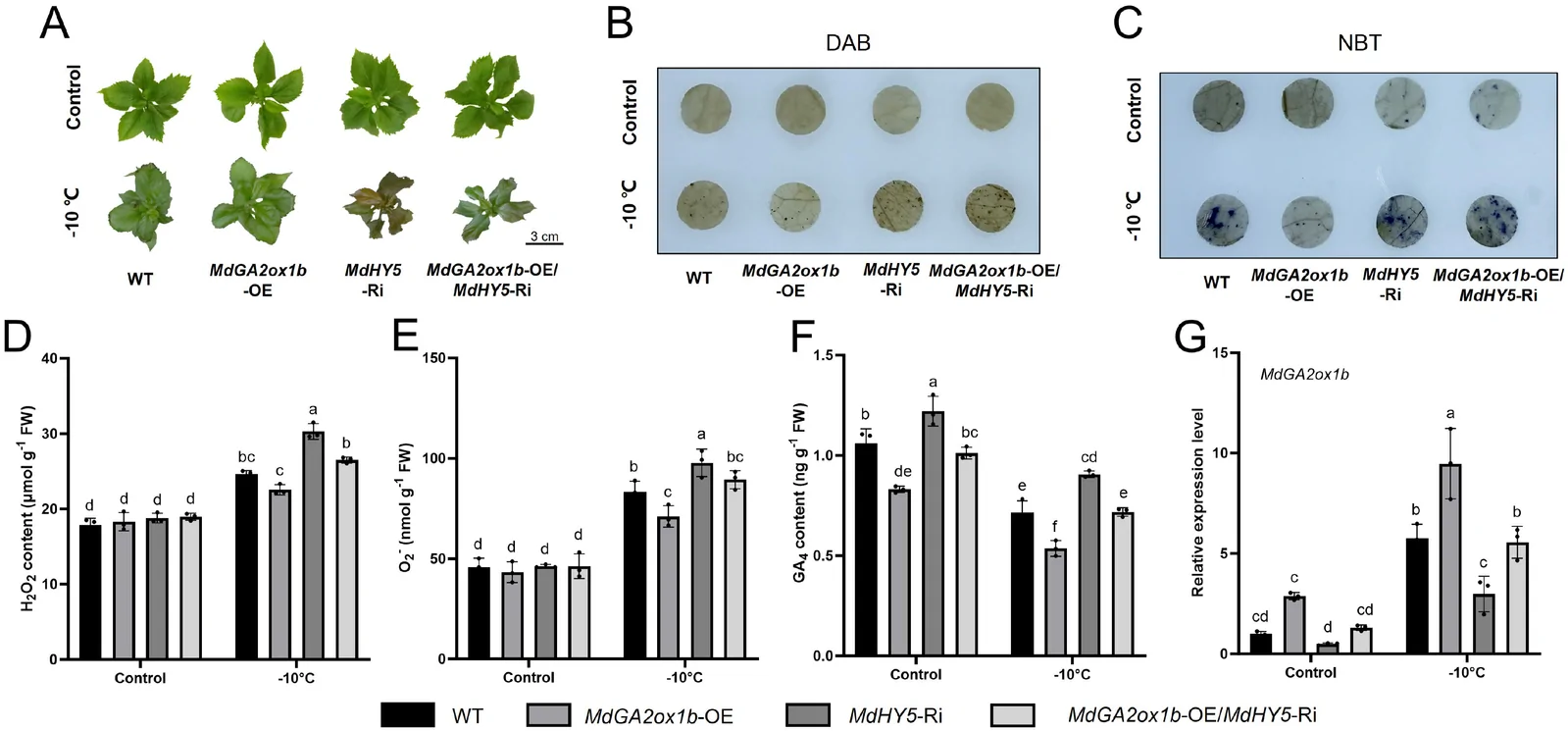
MdHY5-mediated regulation of MdGA2ox1b contributes to cold tolerance in apple. **(A)** Phenotypes of WT, MdGA2ox1b-OE, MdHY5-Ri, and MdGA2ox1b-OE/MdHY5-Ri apple leaves under control and −10°C conditions. **(B, C)** DAB and NBT staining, respectively, showing reactive oxygen species accumulation in the indicated genotypes under control and cold-stress conditions. **(D, E)** Quantification of H₂O₂ and O₂⁻ contents, respectively. **(F)** GA₄ content. **(G)** Relative expression level of MdGA2ox1b. Different letters indicate significant differences among treatments.

The evolution of apple *Md2OGD-Hs* was predominantly driven by WGD rather than tandem duplication, which is consistent with the paleopolyploidization history of Rosaceae plants (Xiang et al., 2017; Wu et al., 2020). First, the Ks peak of the 34 segmental duplication pairs in the *Md2OGD-H* family was 0.2344, closely matching the apple whole-genome Ks peak at 0.249, corresponding to the lineage-specific WGD event dated to 30–45 million years ago (Velasco et al., 2010; Daccord et al., 2017). Second, 39 of the 58 *Md2OGD-H* genes, accounting for 67.24% of the family, were located within WGD-derived collinear blocks, significantly exceeding the genome-wide average of 52.96%, with chi-square test giving χ² = 4.187, *p* = 0.041, and OR = 1.823. Third, Ka/Ks ratios for all 34 duplication pairs ranged from 0.12 to 0.46, all far below 1, indicating purifying selection and functional maintenance. Notably, the expansion pattern of Md2OGD-Hs differs markedly from that in other eudicots. In tomato, approximately 28% of *Sl2OGD-H* members originated from WGD, with dispersed duplication being the predominant mechanism (Ding et al., 2020). In grape, about 30% of *Vv2OGD-H* genes arose from WGD, while tandem and proximal played a major role (Gao et al., 2022). By contrast, our analysis shows that 67.2% of *Md2OGD-H* genes in apple are derived from WGD. This prominent contribution of WGD to the Md2OGD-H family is consistent with the lineage-specific paleopolyploidization history of apple, which sets it apart from species that have not experienced recent WGD events. Interestingly, the subclades of *C19-GA2oxs* and *C20-GA2oxs* accounted for the highest proportions of segmental duplication events (**Figure 3**; **Supplementary Table S5**). We propose two driving forces for this preferential retention. First, substrate division: C19-GA2ox and C20-GA2ox target different GA precursors, with duplicates undergoing subfunctionalization after WGD. Second, perennial stress adaptation: multiple GA2ox copies provide buffering capacity for rapid GA inactivation, enabling a dynamic growth–defense trade-off in apple. Additionally, cross-species synteny analysis revealed conserved colinearity between apple and distantly related eudicots, unlike monocots, which showed stark reductions (**Figure 4**; **Supplementary Table S6**), hence supporting rapid functional divergence of *2OGD-Hs* post-eudicot-monocot split (Ma et al., 2023).

*MdGA2ox1b* showed peak expression in flowers (**Supplementary Figure S3**), suggesting that the GA catabolism regulates floral development or fruit set (Zhang et al., 2023a). Further, transgenic calli assays confirmed that *MdGA2ox1b* positively regulates salt, cold and heat tolerance through enhanced ROS scavenging (**Figure 7**).

## 5 Conclusion

This study systematically identified 257 *Md2OGD* genes in apple, including 58 *Md2OGD-H* genes involved in hormone biosynthesis, metabolism, and stress responses. Species-specific synteny analysis indicated that WGD primarily drove the expansion of *Md2OGD-Hs*, while inter-species collinearity revealed closer evolutionary relationships with eudicots than monocots. Promoter cis-element analysis highlighted enrichment of light-responsive, hormone-related, abiotic stress-responsive, and developmental regulatory motifs. Expression profiling demonstrated that *MdGA2ox1b* and other members were significantly induced under salt, drought, cold, and heat stress, with most genes dynamically regulating fruit ripening. Moreover, MdHY5 activates the expression of *MdGA2ox1b*, and this activation is further enhanced under stress conditions. This work identifies key candidates that unravel hormone-stress crosstalk in apple and provides a molecular basis for breeding stress-resilient cultivars.

## Data Availability

The original contributions presented in the study are included in the article/**Supplementary Material**. Further inquiries can be directed to the corresponding author.
