# Supplementary material for: Genome-wide characterization of the 2OGD-Hs gene family in apple reveals a GA-metabolic gene, MdGA2ox1b, confers stress tolerance via MdHY5 regulation
Source: Front Plant Sci. 2026 Oct 15;17:1911733. doi: 10.3389/fpls.2026.1911733 (PMC13717247; doi:10.3389/fpls.2026.1911733)
Supplement: Supplementary file 1 [file Supplementaryfile1.docx]

**Figure S1. Phylogenetic tree of Md2OGD proteins identified in Arabidopsis and apple.**

**Figure S2. Structural features of the *Md2OGD-Hs*.**

**Figure S3. Subcellular localization and tissue-specific expression pattern analysis of *MdGA2ox1b*.**

**Figure S4. Identification of *MdGA2ox1b* transgenic apple calli.**

(A) Genomic PCR identification of *MdGA2ox1b*-OE/RNAi apple calli. Marker, DL2000. OE, overexpression; Ri, RNA interfere. (B) RT-qPCR identification of *MdGA2ox1b*-OE/RNAi apple calli. Letters indicate statistically homogeneous groups following a one-way analysis of variance with Tukey’s post hoc test, with *P* < 0.05 as the significance level.

**Fig. S7 Analysis of ROS levels in *MdGA2ox1b*-transgenic apple calli exposed to various stresses.**

(A-B) Contents of H_2_O_2_ and O_2_^-^ in *MdGA2ox1b*-transgenic calli under salt stress. (C-D) Contents of H_2_O_2_ and O_2_^-^ in *MdGA2ox1b*-transgenic calli under cold stress. (E-F) Contents of H_2_O_2_ and O_2_^-^ in *MdGA2ox1b*-transgenic calli under heat stress. Data are means ± SD (n=3). Letters indicate statistically homogeneous groups following a one-way analysis of variance with Tukey’s post hoc test, with *P* < 0.05 as the significance level.

**Fig. S8 Expression levels of genes related to stresses of *MdGA2ox1b* transgenic apple plantlets.**

(A) Gene expression of apple plantlets with transient overexpression and silencing of *MdGA2ox1b* under salt stress. (B) Gene expression of apple plantlets with transient overexpression and silencing of *MdGA2ox1b* under cold stress. (C) Gene expression of apple plantlets with transient overexpression and silencing of *MdGA2ox1b* under heat stress. Data are means ± SD (n=3). Letters indicate statistically homogeneous groups following a one-way analysis of variance with Tukey’s post hoc test, with *P* < 0.05 as the significance level.
